# Knowledge on anaemia and benefit of iron–folic acid supplementation among pregnant mothers attending antenatal care in Woldia town, Northeastern Ethiopia: a facility-based cross-sectional study

**DOI:** 10.1186/s41043-022-00315-9

**Published:** 2022-08-04

**Authors:** Asmamaw Demis Bizuneh, Getnet Gedefaw Azeze

**Affiliations:** 1 School of Nursing, College of Health Sciences, Woldia University, P.O. Box: 400, Woldia, Ethiopia; 2 Present Address: Department of Midwifery, College of Health Sciences, Injibara University, P.O. Box: 40, Injibara, Ethiopia

**Keywords:** Knowledge, Anaemia, Pregnant mother, Antenatal care, Woldia town

## Abstract

**Background:**

Anaemia in pregnancy is the leading cause of maternal morbidity and mortality and poor birth outcomes in low- and middle-income countries. The most common cause of anaemia during pregnancy is acute blood loss and iron deficiency due to physiological changes and increasing demand for iron on the mother and growing foetus. Iron and folic acid supplementation is the most widely employed strategy to alleviate iron deficiency anaemia during pregnancy. The mother’s knowledge of anaemia and the benefit of iron–folic acid is crucial in reducing the magnitude of anaemia due to iron deficiency. In Woldia town, despite the efforts made to reduce iron deficiency anaemia during pregnancy, information on pregnant mother knowledge on anaemia and the benefit of iron–folic acid and its associated factors are scarce.

**Methods:**

A facility-based cross-sectional study design was conducted, on 414 pregnant mothers attending antenatal care in Woldia town, Northern Ethiopia. Systematic random sampling methods were used to select study participants. The data were entered into Epi-data version 4.2 and analysed using SPSS version 24. Bivariable and multivariable analysis was done to see the association between the dependent variable and independent variables.

**Results:**

This study revealed that 54.1% and 57.7% of pregnant women had good knowledge of anaemia and the benefit of iron–folic acid, respectively. Maternal education status (AOR = 2.19, 95% CI 1.32–3.64), good knowledge of iron–folic acid (AOR = 5.85, 95% CI 3.60–9.50) and residence (AOR = 5.43, 95% CI 2.36–12.51) were statistically associated with pregnant mothers knowledge on anaemia. Obtained counselling on the benefit of iron–folic acid (AOR = 2.04, 95% CI 1.11–3.75), having four or more antenatal care visit (AOR = 3.12, 95% CI 1.38–7.07) and good knowledge of anaemia (AOR = 5.88, 95% CI 3.63–9.50) was statistically associated with pregnant mothers knowledge on the benefit of iron–folic acid.

**Conclusions:**

Promoting frequent antenatal care visits and giving counselling on the benefit of iron–folic acid and cause, prevention and treatment of anaemia were essential strategies to raise knowledge of pregnant mother on anaemia and the benefit of iron–folic acid.

## Background

Anaemia is a global public health problem affecting pregnant mothers resulting in maternal mortality and morbidity and poor birth outcome [1]. Globally, anaemia affects around two billion people and approximately half of all anaemia can be attributed to iron deficiency [2]. It is estimated that 38% of pregnant women worldwide are anaemic with the highest in Africa followed by South East Asia which accounts for 62.3% and 53.8%, respectively [3, 4]. According to the World Health Organization (WHO) report, 3.7% of maternal mortality in Africa is directly attributed to anaemia [5]. In Ethiopia, the prevalence of anaemia in pregnant women is reported to be29% which is a major public health problem [6]. Different studies conducted in Ethiopia also showed that the prevalence of anaemia among pregnant women was ranged from 20 to 60% [7–13]. Iron supplementation is the most widely employed strategy to alleviate iron deficiency anaemia both globally and nationally [14, 15]. World Health Organization and National guideline current recommendation on the treatment of anaemia during pregnancy include improvements in dietary diversity; food fortification with iron, folic acid and other micronutrients; and daily supplementation of iron and folic acid to each pregnant women and control of infections [15, 16]. In Ethiopia, nationally only 5% of pregnant women took iron–folic acid supplementation (IFAS) for greater than 90 days, but 58% did not take any iron–folic acid tablets during their most recent pregnancy [6]. A study on the importance of antenatal use of iron and folic acid supplement showed that it can eliminate 50% of iron deficiency anaemia in pregnant women [17]. Pregnant women in developing countries are at risk of anaemia due to poverty, grand multiparty, too early pregnancies, too many and too frequent pregnancies spacing of < 1 year, low socioeconomic status, illiteracy and late booking of pregnant women at antenatal care units [11, 12, 18]. Many of these risk factors can be minimized if the mother knowledge of the cause and prevention of anaemia will be improved. Despite anaemia having been identified as a global public health problem for several years, no rapid progress has been observed, and the prevalence of the disease is still high globally and locally. Very few researches [19, 24] are done in Ethiopia regarding knowledge of anaemia and the benefit of iron–folic acid supplementation in pregnant mothers. Therefore, this study assessed knowledge on anaemia and the benefit of iron–folic acid supplementation and associated factors among pregnant mothers attending antenatal care in public health institutions of Woldia town, North-eastern Ethiopia.

## Methods

### Study setting and period

The study was conducted from February to March 2018 at public health institutions in Woldia town, North Wollo zone. Woldia is the capital city of the North Wollo Zone which is located about 521 kms from Addis Ababa, the capital city of Ethiopia, 358 km south-east from Bahirdar city of Amhara regional state. It is located North of Dessie and South-east of Lalibela in the Amhara region. It is situated with an elevation of 2112 m above sea level. The town has four rural and three urban kebeles. The estimated total population of the town is 71,460 of which male populations constitute 35, 397 (49.53%) and female accounts for 36, 063 (50.47%). Out of the total population, 18,356 (25.69%) of them were in the reproductive age group. Regarding health services, the town comprises governmental (one regional hospital, two health centres, four health posts), non-governmental (five small and eight medium private clinics, two private drug vendors and six private drugstores), non-profit non-governmental (Family Guidance Associations).

### Study design and participants’ characteristics

A facility-based quantitative cross-sectional study design was implemented. All pregnant mothers who were attending antenatal care services in Woldia town public health facilities were the source population, and all systematically selected pregnant women who were attending antenatal care in the selected facilities during the study period were included in the study. Seriously ill mothers were excluded from the study.

### Sample size determination and sampling procedure

The sample size was determined by a computer-based on Epi info 7 software Stat Cal using single population proportion formula.

The actual sample size is calculated using a single population proportion formula.

$$n = \frac{{\left( {Z_{\alpha /2} } \right)^{2} p\left( {1 - P} \right)}}{{d^{2} }} = \frac{{\left( {1.96} \right)^{2} *0.573\left( {1 - 0.573} \right)}}{{\left( {0.05} \right)^{2} }} = 376$$

where *n* = minimum sample size required for the study, *Z* = standard normal distribution (*Z* = 1.96) with a confidence interval of 95%, and *P* = knowledge of pregnant women on anaemia in West Shoa Zone Ethiopia (*p* = 57.3%) [19]. *d* = is a tolerable margin of error (*d* = 0.05).

The final sample size was come up by adding a non-response rate of 10% to the sample size from 376. So, the final sample size for the study is 414.

In Woldia Town Administration, there were two public health centres and one General hospital. All of those facilities were included in the study. The average estimated number of pregnant women attending the antenatal clinic in each antenatal facilities for six months was taken. The total estimated number of pregnant women attending antenatal clinic in each antenatal institution for a single month was taken (786), and sampling with population proportional to size (PPS) was calculated for each institution to give the total sample size by using the following formula. Accordingly, the calculated sample was distributed into these health facilities proportional to the size of women attending antenatal care. Finally, a systematic random sampling technique was used to include participants in the study.

$$\begin{aligned} & {\text{The sample size for each health facility}} \\ & \quad = \frac{{{\text{No of respondents from each health facility }} \times {\text{ final sample size}}}}{{\text{Total number of the source population}}} \end{aligned}$$

Woldia General Hospital $\mathrm{ni}=\frac{342}{786}*414 = 180$ respondents were selected.

Woldia Health Center $\mathrm{ni}=\frac{281}{786}*414 = 148$ respondents were selected.

Gonder Ber Health Center $\mathrm{ni}=\frac{163}{786}*414 = 86$ respondents were selected.

### Method of data collection

Information on socio-demographic factors, obstetrics-related factors and knowledge-related factors was collected by face-to-face interview. The knowledge assessment tool was adapted from a study done in Kenya and South Ethiopia [19–21, 23]. Knowledge of anaemia was assessed by 25 reliable items (Cronbach’s alpha = 0.79) (items on ever heard anaemia, sign symptom, cause, consequences and prevention of anaemia), and knowledge of the benefit of iron–folic acid supplementation was assessed by nine reliable items (Cronbach’s alpha = 0.75) (items on the benefits of IFAS and frequency and duration of taking IFA supplements). The maternal charts were reviewed to collect information regarding some variables like haemoglobin value, previous history of chronic illness, maternal anaemia.

### Data quality assurance

The training was given to data collectors and supervisors about techniques of data collection and briefed on each question included in the data collection tool. The pre-test was conducted among 5% of the population to ensure the validity of the tool, and then, the correction was made before the actual data collection. The English language questionnaire was translated into the Amharic language (a language spoken in the study area) and was translated back to the English language, and a comparison was made on the consistency of the two versions. The principal investigator and supervisors were checked on the spot and reviewed all the questionnaires to ensure completeness and consistency of the information collected, and immediate action was taken accordingly. To minimize bias, interviews were conducted in an area with adequate confidentiality and privacy. Double data entry was done by two data clerks, and the consistency of the entered data was cross-checked by comparing the two separately entered data.

### Data processing and analysis

The data were coded, cleaned, edited and entered into Epi-data version 4.2 and exported to SPSS window version 24 for analysis. Comprehensive knowledge of anaemia was computed from summing up all relevant 25 knowledge items (item on ever heard anaemia, sign symptom, cause, consequences and prevention of anaemia). A correct answer for each item was scored as “1”, and an incorrect answer was scored as “0”. Items were then summed up and converted into 100, and the mean score was calculated. Accordingly, the mean score was 44.8 (SD = 22.6). Finally, those respondents who scored mean and above were labelled as having good knowledge about anaemia. Comprehensive knowledge of iron–foliate supplement was computed by summing up all relevant nine items (items on benefits of iron–foliate supplementation and frequency and duration of taking IFA supplements). A correct answer for each item was scored as “1”, and an incorrect answer was scored as “0”. Items then were summed up and converted into 100, and the median score was calculated. The mean score was 51.6 (SD = 19.8). Finally, those respondents who scored mean and above were labelled as having good knowledge of the benefit of IFAS. All variables with *P* ≤ 0.25 in the bivariate analysis were included in the final model of multivariate analysis to control all possible confounders. Multi-collinearity was checked to see the linear correlation among the independent variables by using standard error. Variables with a standard error of > 2 were dropped from the multivariable analysis. Model fitness was checked with the Hosmer–Lemeshow test. Adjusted odds ratio with 95% CI was estimated to identify the factors associated with adherence status using multivariable logistic regression analysis. The level of statistical significance was declared at a *P* value < 0.05.

## Results

### Socio-demographic characteristics

A total of 414 study participants were involved in the study making a response rate of 100%. The mean age of study participants was 26.35 (± 4.25 SD) years. All most all, 405 (97.8%) of the study participants were married, 294 (71.0%) were Orthodox by religion and 386 (93.2%) were Amhara by ethnicity. One hundred and eleven (26.9%) were at the college level and above, and 187 (45.2%) were housewives. The majority, 359 (86.7%), of the respondents were from urban residents, and 240 (58.0%) had 1–3 family size (Table 1).

**Table 1:** Socio-demographic characteristics of pregnant mother’s attending antenatal care in public health facilities of Woldia Town, North-eastern Ethiopia, 2018

Variables	Frequency	Percentage (%)
Age		
15–19	14	3.4
20–24	130	31.4
25–29	183	44.2
30–34	62	14.9
≥ 35	25	6.1
Marital status		
Married	405	97.8
Others*	9	2.2
Religion		
Orthodox	294	71.0
Muslim	108	26.1
Others**	12	2.9
Ethnicity		
Amhara	386	93.2
Others***	28	6.8
Educational level		
Can’t read and write	79	19.1
Can read and write	85	20.5
Primary education (grade 1–8th)	61	14.7
Secondary education (grade 9–12th)	78	18.8
College and above	111	26.9
Occupation		
Housewife	187	45.2
Govt employee	98	23.7
Self-employed	68	16.4
Farmer	42	10.1
Others****	19	4.6
Family size		
1–3	240	58.0
≥ 4	174	42.0
Place of residence		
Urban	359	86.7
Rural	55	13.3

Other*: Divorced. Widowed, Single, Other**: Protestant, Catholic, Other***: Afar, Oromo, Tigray, Other****: Student, Daily labourer, Private employee

### Obstetrics-related characteristics

More than half, 218 (52.7%), of pregnant mothers were in the third trimester, and 237 (57.2%) were early registered for ANC (< 16 weeks). Regarding gravidity and parity, more than two-third, 278 (67.1%), of women were multigravida and 184 (44.4%) were primiparous. This study showed that only 33 (7.9%) of women had a history of anaemia in the current pregnancy. Concerning the utilization of antenatal care, the majority, 356 (85.9%), of respondents had less than four ANC visits and 380 (91.8%) of respondents did not have any previous medical illness (Table 2).

**Table 2:** Obstetrics-related characteristics of pregnant mother’s attending antenatal care in public health facilities of Woldia Town, North-eastern Ethiopia, 2018

Variables	Knowledge of anaemia		Knowledge of IFAS	
	Good knowledge (%)	Poor knowledge (%)	Good knowledge (%)	Poor knowledge (%)
Gravidity				
Primigravida	61 (44.9)	75 (55.1)	64 (47.1)	72 (52.9)
Multigravida	163 (58.6)	115 (41.4)	175 (62.9)	103 (37.1)
Parity				
Nulliparous	76 (55.5)	61 (44.5)	73(53.3)	64(46.7)
Primiparous	97 (52.7)	87 (47.3)	115(62.5)	69(37.5)
Multiparous	51 (54.8)	42 (45.2)	51(54.8)	42(45.2)
Birth Spacing in years (*n* = 277)				
1–2	23 (41.1)	33 (58.9)	26(46.4)	30(53.6)
≥ 3	125 (56.6)	96 (43.4)	140(63.3)	81(36.7)
ANC visit				
≥ 4 visits	42 (72.4)	16 (27.6)	45 (77.6)	13 (22.4)
< 4 visits	182 (51.1)	174 (48.9)	194 (54.5)	162 (45.5)
First registration time for ANC				
Early registration	142 (59.9)	95 (40.1)	148 (62.4)	89 (37.6)
Late registration	82 (46.3)	95 (53.7)	91 (51.4)	86 (48.6)
Medical illness other than anaemia				
Yes	19 (55.9)	15 (44.1)	17 (50.0)	17 (50.0)
No	205 (53.9)	175 (46.1)	222 (58.4)	158 (41.6)
History of anaemia during a previous pregnancy (*n* = 277)				
Yes	18 (54.5)	15 (45.5)	19 (57.6)	14 (42.4)
No	129 (52.9)	115 (47.1)	146 (59.8)	98 (40.2)
Current anaemia status				
Anaemic	25 (71.4)	10 (28.6)	26 (74.3)	9 (25.7)
Non-anaemic	199 (52.5)	180 (47.5)	213 (56.2)	166 (43.8)
Obtain counselling on IFA				
Yes	200 (57.8)	146 (42.2)	221 (61.6)	138 (38.4)
No	24 (35.3)	44 (64.7)	18 (32.7)	37 (67.3)

### Knowledge of pregnant mothers on anaemia and benefit of iron–folic acid supplementation

More than half, 224 (54.1%), of the respondents had good knowledge about anaemia, whereas 239 (57.7%) of the respondents had good knowledge about the benefit of iron and folic acid supplementation***.***

### Factors associated with knowledge of anaemia

The covariates of this study were: the mother’s educational status, residence, having anaemia in the current pregnancy, gravidity, ANC visit, first registration week, obtained counselling about IFAS and knowledge of the benefit of IFAS were a candidate for the multivariable model. In the multivariable model maternal educational status, knowledge of IFAS and residence were statistically associated with knowledge of anaemia.

Mothers who were from urban residents were 5.4 times more likely knowledgeable about anaemia than mothers who were from rural residents (AOR = 5.43, 95% CI 2.36–12.51). Mothers who had completed secondary education and above were two times more likely knowledgeable about anaemia than mothers who attended non-formal education (AOR = 2.19, 95% CI 1.32–3.64). Regarding knowledge of IFAS, those who had good knowledge on benefit IFAS were almost six times (AOR = 5.85, 95% CI 3.60–9.50) more likely knowledgeable to anaemia as compared to those who had poor knowledge of IFAS (Table 3).

**Table 3:** Factors associated with knowledge of anaemia among pregnant mother’s attending antenatal care in public health facilities of Woldia town, North-eastern Ethiopia, 2018

Variables	Knowledge of anaemia		COR (95%CI)	AOR (95%CI)
	Good (%)	Poor (%)		
Mother educational status				
Non-formal education	69 (41.8)	96 (58.2)	1	1
Primary (Grade 1–8th)	31 (51.7)	29 (48.3)	1.48(0.82–2.69)	2.34(1.14–4.82)*
Secondary and above	124 (65.6)	65 (34.4)	2.65(1.72–4.08)	2.19(1.32–3.64)**
Residence				
Urban	215 (59.9)	144 (40.1)	7.63 (3.62–16.07)	5.43 (2.36–12.51)***
Rural	9 (16.4)	46 (83.6)	1	1
Gravidity				
Multigravida	149 (53.6)	129 (46.4)	1.74 (1.15–2.63)	1.35 (0.82–2.23)
Primigravida	75 (55.1)	61 (44.9)	1	1
Current anaemia status				
Anaemic	25 (71.4)	10 (28.6)	2.26 (1.05–4.84)	1.84 (0.76–4.41)
Non-anaemic	199 (52.5)	180 (47.5)	1	1
Obtain counselling on IFA				
Yes	200 (57.8)	146 (42.2)	2.51 (1.46–4.31)	1.64 (0.86–3.11)
No	24 (35.3)	44 (64.7)	1	1
Frequency of ANC visits				
≥ 4 visits	46 (79.3)	12 (20.7)	3.83 (1.96–7.48)	1.97 (0.92–4.22)
< 4 visits	178 (50.0)	178 (50.0)	1	1
Time of registration				
Early (< 16 weeks)	142 (59.9)	95 (40.1)	1.73 (1.17–2.56)	1.35 (0.83–2.18)
Late (≥ 16 weeks)	82 (46.3)	95 (53.7)	1	1
Knowledge of IFAS				
Good knowledge	176 (73.6)	63 (26.4)	7.39 (4.76–11.47)	5.85 (3.6–9.50)***
Poor knowledge	48 (27.4)	127 (72.6)	1	1

Significant at: *P = 0.021, **P = 002, ***P < 0.001, 1 = constant

### Factors associated with knowledge on the benefit of iron–folic acid supplementation

The covariates of this study were: mother’s education, residence, current anaemia, ANC visit, first registration week, obtained counselling about IFAS and knowledge of anaemia were a candidate for the multivariable model. In the multivariable model ANC visit, knowledge of anaemia and obtained counselling about IFAS was statistically associated with knowledge on the benefit of IFAS. Mothers who had obtained counselling about IFAS were two times more likely knowledgeable on the benefit of IFAS than mothers who had not obtained counselling (AOR = 2.04, 95% CI 1.11–3.75). Mothers who had four or more ANC visit were three times (AOR = 3.12, 95% CI 1.38–7.07) more likely knowledgeable on the benefit of IFAS than mothers who had less than four times ANC visit. Regarding knowledge of anaemia, those who had good knowledge of anaemia were almost six times (AOR = 5.88, 95% CI 3.63–9.50) more likely knowledgeable on the benefit of IFAS as compared to those who had poor knowledge of anaemia (Table 4).

**Table 4:** Factors associated with knowledge on the benefit of IFAS among pregnant mother’s attending antenatal care in public health facilities of Woldia town, North-eastern Ethiopia, 2018

Variables	Knowledge of IFAS		COR (95%CI)	AOR (95%CI)
	Good (%)	Poor (%)		
Mother educational status				
Non-formal education	89 (53.9)	76 (46.1)	1	1
Primary (Grade 1–8^th^)	25 (41.7)	35 (58.3)	0.61 (0.33–1.11)	0.41 (0.20–1.24)
Secondary and above	125 (66.1)	64 (33.9)	1.66 (1.08–2.56)	1.00 (0.60–1.66)
Residence				
Urban	221 (61.6)	138 (38.4)	3.29 (1.80–6.01)	1.50 (0.76–2.95)
Rural	18 (32.7)	37 (67.3)	1	1
Current anaemia status				
Anaemic	26 (74.3)	9 (25.7)	2.25 (1.02–4.93)	1.72 (0.71–4.16)
Non-anaemic	213 (56.2)	166 (43.8)	1	1
Obtain counselling on IFA				
Yes	214 (61.8)	132 (38.2)	2.78 (1.62–4.77)	2.04 (1.11–3.75)*
No	25 (36.8)	43 (63.2)	1	1
Frequency of ANC visits				
≥ 4 visits	49 (84.5)	9 (15.5)	4.75 (2.26–9.97)	3.12 (1.38–7.07)**
< 4 visits	190 (53.4)	166 (46.6)	1	1
Time of registration				
Early (< 16 weeks)	148 (62.4)	89 (37.6)	1.57 (1.06–2.33)	1.11 (0.69–1.77)
Late (≥ 16 weeks)	91 (51.4)	86 (48.6)	1	1
Knowledge of anaemia				
Good knowledge	176 (78.6)	48 (21.4)	7.39 (4.76–11.47)	5.88 (3.63–9.50)***
Poor knowledge	63 (33.2)	127 (66.8)	1	1

Significant at: *P = 022, **P = 0.006, ***P < 0.001, 1 = constant

## Discussion

In this study, 54.1% of pregnant women had good knowledge of anaemia. This finding was in line with a study conducted in India, (52.5%) [20]**,** the rural district of Ethiopia (51.4%) [21]**.** But it was lower than the study conducted in North Showa Zone, Ethiopia (57.3%) [19], Pune, India (69%) [23], and Tamil Nadu, India (76.5%) [26]**.** The difference might be due to the time gap between the studies and the difference in socio-demographic characteristics of study participants. It was higher than a study conducted in South Ethiopia 44.3% [24] and Egypt at 40% [25]**.** This might be currently due to increase in information access for pregnant mothers, and since the majority of the study participants were from urban residents, being from an urban resident is strongly associated with pregnant mother knowledge of anaemia.

The knowledge of pregnant women about the benefit of iron–folic acid supplementation was found to be 57.7%. This finding was higher than a study conducted in Singur, West Bengal, India (39.9%) [22]**.** This difference might be due to the difference in socio-demographic characteristics of the study participants and the time gap between the studies.

Pregnant mothers who completed secondary education and above were two times more likely knowledgeable about anaemia as compared with those pregnant mothers who attended non-formal education. This is in line with the study conducted in South Ethiopia [24], North Showa Zone, Ethiopia [19]**,** Tamil Nadu, India (76.5%) [26], and Pune, India (69%) [23]**.** This might be due to the fact that as the educational level increases, the ability of the pregnant mother to understand the cause, prevention and treatment of anaemia during counselling session of antenatal care will rise which ultimately increases the knowledge level of pregnant mothers on anaemia.

Pregnant mothers who were from urban residents were five times more likely knowledgeable to anaemia as compared with pregnant mothers who were rural residents. This is supported with the study conducted in North Showa Zone, Ethiopia [19]**.** This might be because pregnant mothers from urban residents will have more information access through mass media and they have more frequent and early antenatal care follow-up which enables them to get more information.

Having good knowledge of the benefit of iron–folic acid supplementation was significantly and positively associated with knowledge of anaemia. This is supported by the study conducted in North Showa Zone, Ethiopia [19]**.** This might be due to the fact that having good knowledge of the benefit of iron–folic acid supplementation helps women to understand the cause, consequence, prevention and treatment of anaemia by taking iron–folic acid which results in good knowledge of anaemia.

Pregnant mothers who had four or more antenatal care visit were three times more knowledgeable as compared with those pregnant mothers who had less than four antenatal care visits. This is supported by a study conducted in South Ethiopia [24], but it is not supported with a study conducted in Pune, India [23]**.** This might be due to the fact that having frequent ANC visits during pregnancy may have a high chance of getting counselling on the benefit of taking iron–folic acid supplementation on prevention of anaemia and neural tube defect which ultimately improves the knowledge on the benefit of iron–folic acid supplementation.

Pregnant mothers who had obtained counselling during ANC visits were two times more likely knowledgeable about the benefit of iron–folic acid supplementation as compared to those pregnant mothers who had not obtained counselling. This is in line with the study conducted in West Bengal, India [22]. This might be due to the fact that pregnant mother who had obtained counselling may understand the benefit of taking the supplement for the pregnant mother and for the growing foetus which ultimately improves their knowledge on the benefit of iron–folic acid supplementation.

### Limitations of the study

The cross-sectional nature of the study design limits the applicability of the findings in establishing causality between the variables, and it might also suffer from recall bias.

## Conclusions

Our data demonstrate that the knowledge level of pregnant mother on anaemia and the benefit of iron and folic acid supplementation was low. Educational status, knowledge of IFAS and residence were independent predictors of pregnant mother knowledge on anaemia, whereas ANC visit, knowledge of anaemia and obtaining counselling about IFAS were independent predictors of pregnant mother knowledge on the benefit of IFAS. We authors would like to recommend during antenatal care follow-up health care providers should counsel pregnant mothers on early frequent ANC visit during pregnancy that focused on anaemia cause, prevention and benefit of taking IFAS in the prevention of anaemia during pregnancy and the postpartum period.

## Data Availability

All related data have been presented within the manuscript. The dataset supporting the conclusions of this article is available from the authors on request.

## Abbreviations

ANC: Antenatal Care
CSA: Central Statistical Agency
EDHS: Ethiopian Demographic and Health Survey
ERC: Ethical Review Committee
FMOH: Federal Ministry of Health
IFAS: Iron and Folic Acid Supplementation
SPSS: Statistical Package for Social Sciences
WHO: World Health Organizations
